# High-intensity focused ultrasound (HIFU) assisted by a rectal Foley catheter for the treatment of recurrent mucinous ovarian cancer: a case report and literature review

**DOI:** 10.3389/fonc.2024.1498631

**Published:** 2024-12-04

**Authors:** Xiaoyin Guo, Wei Liu, Kun Zhou, Hui Zhu, Lu Pan, Chunping Feng, Ling Liu

**Affiliations:** ^1^ Clinical Center for Tumor Therapy, The Second Affiliated Hospital, Chongqing Medical University, Chongqing, China; ^2^ Department of Rehabilitation Medicine, The Second Affiliated Hospital, Chongqing Medical University, Chongqing, China

**Keywords:** high-intensity focused ultrasound, mucinous ovarian cancer, tumor recurrence, chemotherapy, combined therapy

## Abstract

​Mucinous ovarian cancer (MOC) is characterized by high malignancy, poor prognosis and a high recurrence rate. Surgical adjuvant chemotherapy is the main treatment for MOC. The recurrence rate of advanced mucinous ovarian cancer following surgery is significantly high, with limited efficacious treatment options available. Moreover, chemotherapy alone results in low sensitivity in this context. High-intensity focused ultrasound (HIFU) not only efficiently ablates targeted tumor lesions but also elicits an immune response within the body and increases tumor cell susceptibility to drugs, thus increasing therapeutic effectiveness. We report a case of recurrent mucinous ovarian cancer treated with HIFU combined with chemotherapy, which effectively controlled tumor growth and prolonged patient survival. High-intensity focused ultrasound in combination with chemotherapy for the treatment of recurrent and surgically difficult ovarian cancer could provide new treatment strategies.

## Introduction

The mortality rate of ovarian cancer (OC), a type of gynecologic malignant tumor, is the highest; approximately 70% of patients with OC within 3 years after first-line therapy relapse. MOC represents a rare subtype of epithelial ovarian carcinoma (EOC), constituting approximately 3% of all EOC cases. In contrast to the more prevalent high-grade serous carcinoma (HGSC), MOC has distinct characteristics in terms of its natural history, molecular profile, chemosensitivity, and prognosis. The prognosis is favorable in the early stage of the disease, whereas patients with advanced and recurrent disease have an exceedingly dismal prognosis (1).

Recurrent ovarian cancer (ROC) often presents with multiple metastases to the upper abdomen and pelvic organs, and imaging examination suggests a mass. Recurrent ovarian cancer patients typically require secondary cytoreductive surgery (SCS) to achieve maximal tumor cell removal, minimize residual lesions, and potentially achieve R0 resection (complete removal of all visible tumors) for optimal tumor reduction. Simultaneously, other systemic treatments are employed to manage the disease (2). HIFU, a noninvasive thermal ablation method, offers unique advantages, particularly for patients who are not eligible for reoperation, by minimizing the number of tumor cells and reducing the tumor burden. HIFU can effectively ablate multiple metastatic lesions in the pelvic region that are challenging to operate on or cannot be surgically resected, thereby achieving the goal of reducing and eliminating tumor cells.

Previous studies have investigated the use of HIFU in treating benign and malignant pelvic solid tumors, including uterine fibroids, ovarian cancer, cervical cancer, endometrial cancer, and rectal cancer (3). The treatment principle involves utilizing the thermal, cavitation, and mechanical effects of ultrasound to induce coagulation necrosis in the targeted tumor area, thereby rendering it inactive. This noninvasive approach offers high precision and conformal ablation for tumor treatment. Furthermore, HIFU can stimulate the body’s immune response and enhance drug sensitivity in tumor cells, thereby increasing therapeutic efficacy (4, 5).

However, lesions located on the posterior wall of the uterus or anterior rectum may extend beyond or partially exceed the therapeutic focal length of the HIFU transducer. To address this limitation without causing damage to surrounding tissues during HIFU ablation therapy, we employed a Foley catheter inserted into the rectum along with water injection to alter lesion positioning through compression via a water-filled sac to achieve optimal therapeutic range coverage via HIFU treatment (6, 7). This approach effectively safeguards surrounding tissues and organs in the posterior field of the target tissue, thereby minimizing complications.

In this study, we present a case of recurrent mucinous ovarian cancer with multiple pelvic metastases that were considered unresectable. Our treatment approach involved combining HIFU with chemotherapy; however, because the tumor’s anterior location relative to the rectum and some lesions being beyond the treatment range of HIFU, we utilized an intrarectal Foley catheter to manipulate the positional relationship of the lesions to achieve successful lesion ablation. This approach also provides valuable insights for managing cases of recurrent ovarian cancer where surgical intervention is not feasible.

## Case report

The patient, a 33-year-old female, was admitted to the hospital on October 13, 2023, where she presented with abdominal pain and a pelvic mass. After relevant examinations, she was diagnosed with ovarian cancer. On October 17, 2023, the patient underwent extensive surgical procedures, including abdominal hysterectomy, bilateral salpingo-oophorectomy, omentum resection, pelvic and abdominal tumor resection, and sigmoid colectomy, as well as the release of adhesions involving the pelvic ureter, bladder and intestine. Postoperative pathology revealed moderately or poorly differentiated mucinous adenocarcinoma in the left fallopian tube and ovary, whereas no involvement was observed in the right fallopian tube or ovary. Infiltration was detected in the intestinal wall), utero-rectal pouch, perirectal and omental tissues. Additionally, proliferative endometria with endometrial polyps, uterine leiomyoma, and chronic inflammation of the cervical mucosa were found. Immunohistochemical analysis revealed negative expression of ER (-), PR (-), P16 (1+), PAX8 (-), CK20 (-), SATB2 (-), CDX2 (-), and WT-1 (-). Villin (1+) and CA125(1+) exhibited weak staining, while P53 was positive in approximately 20% of the cells, along with a high Ki-67 proliferation index (70%). The tumor multigene high-throughput sequencing test (large panel) conducted on November 9, 2023, did not detect any clinically significant variants (BRAF, BRCA1, BRCA2, NTRKI, NTRK2, NTRK3, RET, TP53, KRAS, PIK3CA, or ERBB2); TMB: 4.2 mutations/Mb; MSS status confirmed.

**Figure 1:**
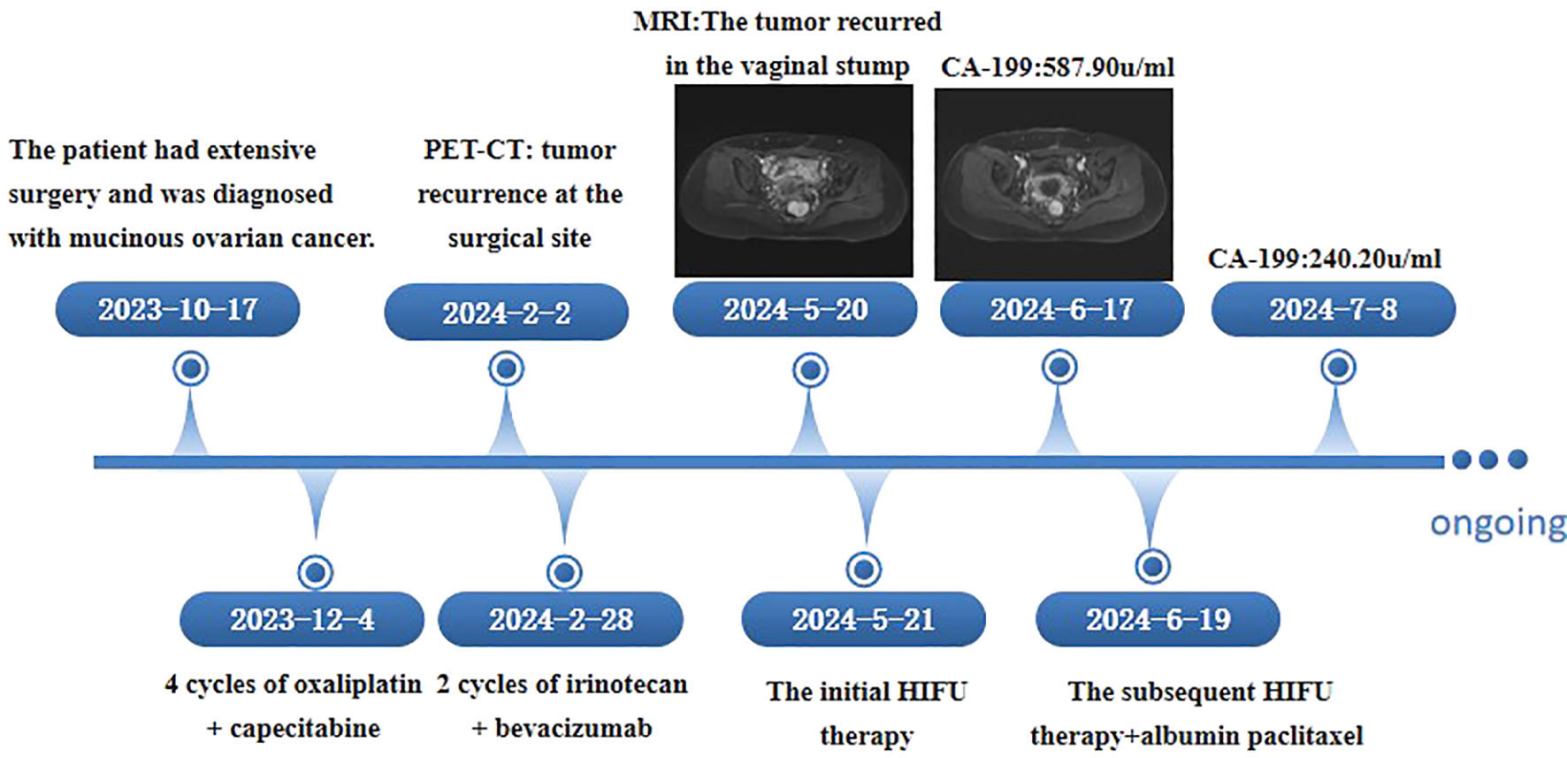
The chronological record of patient assessment and therapeutic intervention.

Four cycles of oxaliplatin plus capecitabine chemotherapy were initiated on December 4, 2023. On February 2nd, 2024, a PET−CT scan indicated tumor recurrence at the surgical site. Two cycles of irinotecan plus bevacizumab were administered on February 28, 2024. Pelvic magnetic resonance imaging (MRI) performed on May 20, 2024, revealed a mass shadow at the vaginal stump, suggesting recurrence. Bilateral pelvic wall lymph nodes were increased in size, indicating metastasis. HIFU was administered under general anesthesia for the treatment of secondary pelvic malignant tumors on May 21, 2024. On May 23, 2024, pelvic magnetic resonance examination revealed extensive necrosis of the vaginal stump lesion, swelling of the surrounding soft tissue, and necrosis of some lymph nodes in the bilateral pelvic wall and presacral space. A follow-up pelvic MRI on June 17, 2024, revealed no change in the overall range of the vaginal stump lesion compared with that on May 23, 2024, with a slightly smaller range of internal nonenhanced necrosis. On June 19, 2024, a second HIFU treatment was subsequently performed for the secondary malignant pelvic tumor under general anesthesia. During surgery and postsurgery (on June 20, 2024), albumin and paclitaxel at doses of 100 mg and 200 mg, respectively, were administered. The CA-199 level decreased significantly from presurgery (587.90 U/ml on June 17, 2024) to postsurgery (240.20 U/ml on July 8, 2024). The chronological record of the patients’ assessments and therapeutic interventions is depicted in **Figure 1**.

## HIFU treatment

The HIFU treatment utilized a JC focused ultrasound tumor therapy system (Chongqing Haifu Medical Technology Co., Ltd., China), which was equipped with a real-time diagnostic ultrasound probe (3.5 MHz to 5.0 MHz), a focal length of 141 mm, a diameter of 120 mm, and a therapeutic transducer with a working frequency of 1.0 MHz. The focus area is an ellipsoid with major and minor axes of 8 mm and 3 mm, respectively.

**Figure 2:**
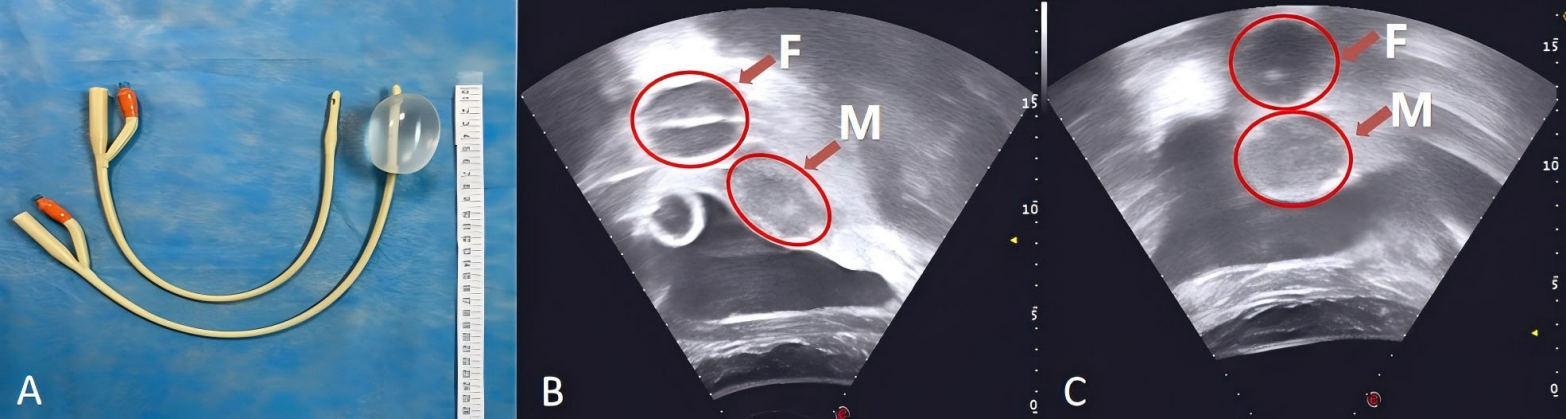
A Foley catheter filled with 60 ml of degassed water **(A)**. The Foley catheter was guided by ultrasound to be placed between the bladder and sacrum via the rectum with degassed water injection **(B, C)**. F, The balloon at the end of the Foley catheter was fixed into the rectum, and degassed water was injected; M, The mass shadow at the vaginal stump.

**Figure 3:**
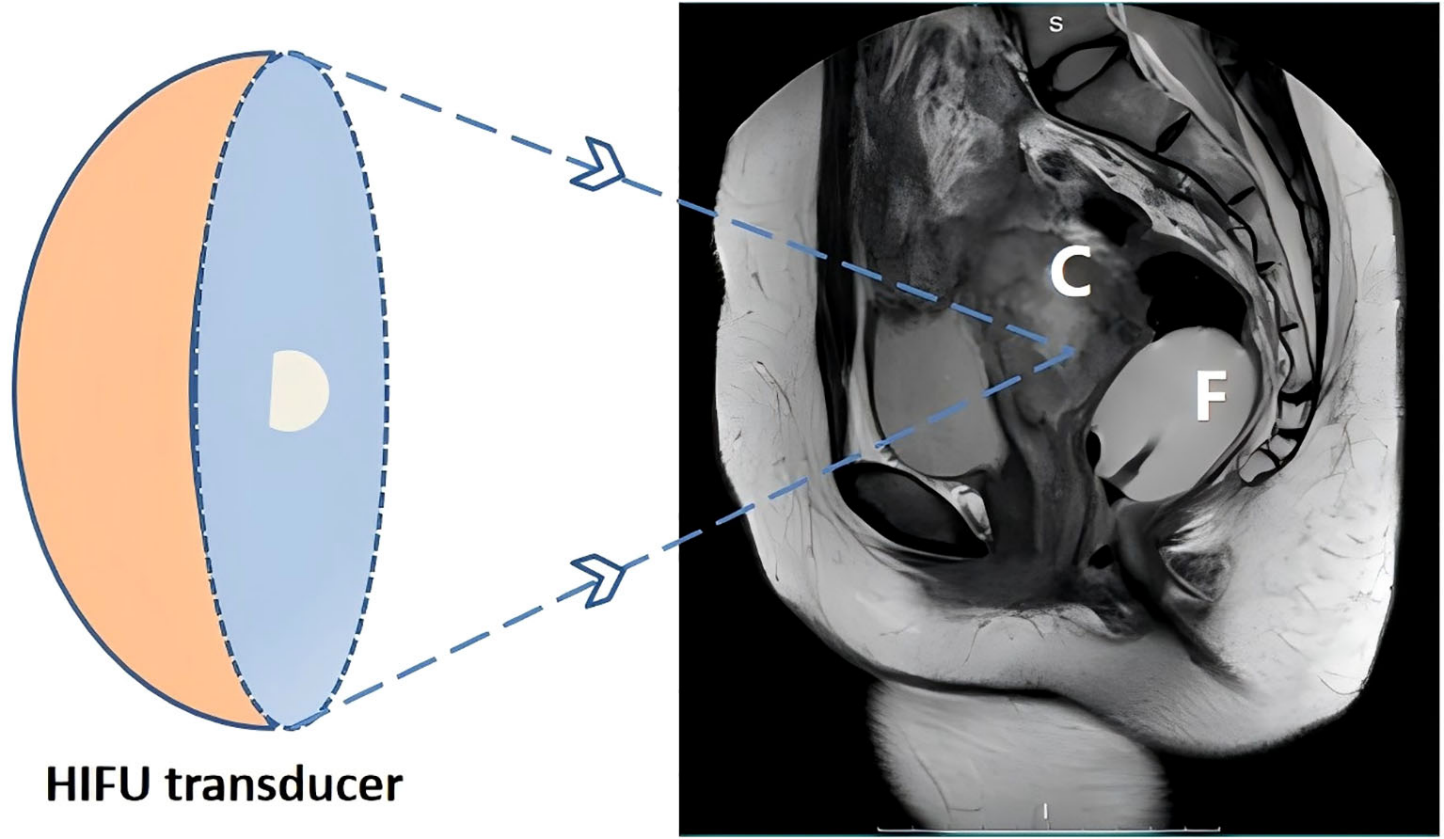
Schematic representation of patient positioning for high-intensity focused ultrasound ablation of ovarian mucinous carcinoma. C, targeted mucinous ovarian cancer; F, The balloon at the end of the Foley catheter was fixed into the rectum, and degassed water was injected; HIFU, high-intensity focused ultrasound.

For HIFU treatment, the patient underwent general anesthesia and was placed in the prone position with a urinary catheter inserted. As the tumor lesion extended beyond the therapeutic range of HIFU, a Foley catheter (Weili Medical Device Co., Ltd., Guangzhou, China) was guided by ultrasound to be placed between the bladder and sacrum via the rectum (**Figure 2**). A nurse checked for integrity, elasticity, and size of the Foley catheter balloon, which was then lubricated with paraffin oil before being placed approximately 8 cm close to the rectal tumor lesion under real-time ultrasound guidance. After the balloon end was secured and filled with degassed water (60 ml), inflation caused the inclusion of the tumor lesion within the HIFU treatment range. A pattern illustrating HIFU treatment for ovarian mucinous carcinoma is shown in **Figure 3**.

**Figure 4:**
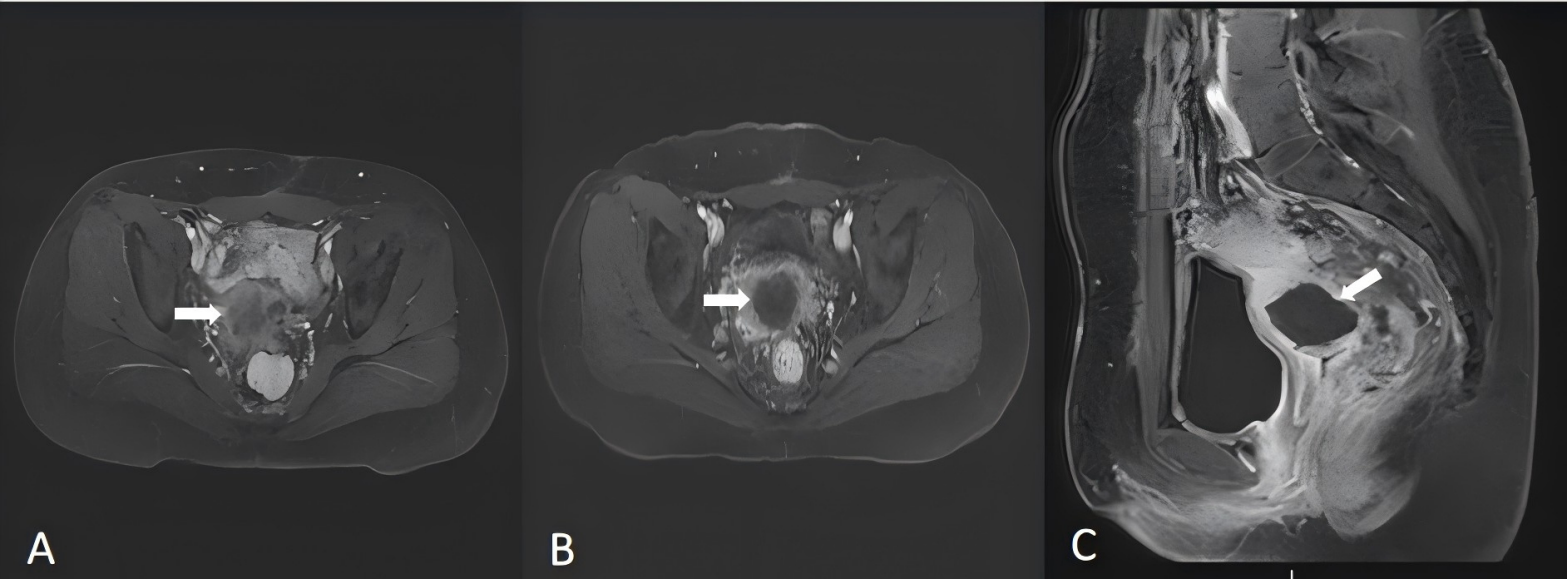
Magnetic resonance imaging (MRI) (2024-5-20) revealed a mass shadow at the vaginal stump, suggesting recurrence **(A)**. Follow-up MRI after HIFU (2024-5-23) revealed that there was no enhancement area in the lesion, which achieved the ablation effect **(B, C)**. The arrows indicate the tumor lesions in the pelvis.

On the basis of the patient’s physical examination and clinical imaging results, a customized HIFU treatment plan was designed. Real-time ultrasound monitoring was utilized to accurately identify tumor lesions and determine the target area for treatment. During HIFU therapy, spot scanning was mainly performed at 100 W to 400 W, with an average power of 250 W and a total treatment time of 600 seconds. Ablation proceeded sequentially from the deepest layer to the superficial layer until complete coverage of the tumor area demonstrated massive grayscale changes (MGSCs).Postoperative imaging data confirmed the successful ablation of the tumor lesions (**Figure 4**).

## Discussion

Ovarian cancer ranks as the third most prevalent malignancy in the female reproductive system, yet it has the highest mortality rate. Approximately 70%-80% of ovarian cancer cases are diagnosed at an advanced stage (8). The incidence and mortality rates of ovarian cancer in China are projected to rise between 2020 and 2030. Ovarian mucinous carcinoma (MOC) represents a rare subtype of epithelial ovarian cancer that differs from serous ovarian cancer (SOC) in terms of occurrence, progression, histopathological characteristics, and clinical features. Owing to distinct treatment modalities, careful discrimination is necessary (9). Currently, treatment options for MOC include surgery, chemotherapy, targeted therapy, and immunotherapy. Surgical intervention combined with adjuvant chemotherapy constitutes the current standard approach for managing MOC patients. Advanced-stage MOC patients (FIGO stage III/IV) undergo cytoreductive surgery; however, owing to its low prevalence, conducting clinical trials becomes challenging, resulting in limited evidence-based medical data and a lack of consensus regarding intraoperative appendectomy indications and postoperative adjuvant chemotherapy selection criteria (1, 9, 10).

HIFU, a noninvasive thermal ablation technique for tumors, can effectively reduce the tumor burden by targeting tumor cells while minimizing the harm inflicted upon patients. The treatment principle leverages the penetrability and aggregation of ultrasound to precisely target the focal area of the tumor, inducing biological effects such as thermal effects, cavitation effects, and mechanical effects. As a result, the temperature at the focal area rapidly increases to 65°C–100°C, leading to cell death within the targeted region and coagulation necrosis of the tumor lesions. Consequently, thermal ablation of the target tissue is achieved without causing damage to surrounding tissues or acoustic channels outside this focus. Compared with other local ablation methods, such as radiofrequency ablation (RFA) and irreversible electroporation (IRE), HIFU has enhanced efficacy in reducing potential complications related to puncture procedures, specifically addressing issues of bleeding and the development of metastatic puncture channels (11).

HIFU has demonstrated favorable clinical outcomes in the treatment of solid tumors, including uterine fibroids, adenomyosis, liver cancer, pancreatic cancer, and osteosarcoma. Moreover, HIFU can induce a thermal effect to increase blood flow within tumor tissue and improve the permeability of tumor cell membranes. Additionally, ultrasonic cavitation and other physical effects can alter molecular structures and facilitate drug uptake while increasing the sensitivity of tumor cells to chemotherapy drugs. Consequently, combining HIFU with chemotherapy can effectively target poorly perfused and quiescent cells to enhance therapeutic efficacy (12). Furthermore, emerging evidence suggests that HIFU may convert immune-cold tumors into immune-hot tumors by enhancing immunotherapy responsiveness (4, 5, 12). In summary, HIFU has synergistic potential in combination therapy.

Owing to the influence of focal length on HIFU treatment efficiency, it is crucial to consider deep pelvic tumors located near intestinal tissues as well as bones and nerves during treatment. This approach helps prevent pain caused by stimulation of the pelvic nerve plexus while reducing the risk associated with intrapelvic fascia swelling or heat deposition-related sacral injuries (6, 13, 14). Therefore, for tumor lesions beyond the focal length range suitable for HIFU treatment but close to the sacrococcygeal region—building upon our previous study utilizing rectal Foley catheterization for treating benign uterine diseases outside this range—we employed a Foley catheter implanted in the rectum along with balloon fixation and degassed water injection to fill the intestinal cavity and ensure proper positioning of tumors within reach for effective HIFU treatment. This can increase the distance between the sacrococcygeal region and the treatment focus area, maintaining a safe distance between the ultrasound energy and the rectum, thereby avoiding the concentration of ultrasound energy in the tumor target area and its absorption by nerves and bones. Consequently, this reduces pain caused by sacrococcygeal nerve stimulation (7). The patient did not experience discomfort associated with placing a Foley catheter in the rectum after HIFU, such as pain or emotional distress. Our previous study validated the safety and feasibility of the use of high-intensity focused ultrasound (HIFU) in conjunction with an intrarectal Foley catheter for the treatment of tumors located beyond the intended treatment area while effectively safeguarding tissues and organs within the posterior field of the target tissue and minimizing complications (7). Moreover, further technical advancements are imperative for enhancing both treatment efficiency and accuracy within the realm of focused ultrasound oncology systems, encompassing the development of transducers featuring extended focal lengths as well as the integration of robot-assisted therapy systems.

There are no existing reports on HIFU treatment for ovarian mucinous carcinoma. In this case, extensive surgical resection confirmed the diagnosis of ovarian mucinous adenocarcinoma. The guidelines provided by the National Comprehensive Cancer Network (NCCN) and genetic testing results do not support the prioritization of targeted therapy and immunotherapy. High Ki-67 expression also indicates increased tumor cell proliferation, enhanced malignancy, and an unfavorable prognosis. However, at less than 4 months postsurgery, rapid tumor recurrence occurred along with disease progression despite the administration of chemotherapy. Owing to extensive pelvic metastasis and a high risk of postoperative recurrence with significant trauma involved during surgery, we combined HIFU therapy with chemotherapy, resulting in coagulative necrosis in most tumors, leading to a slower tumor growth rate and prolonged patient survival time. CA-199 is a sensitive marker for MOC diagnosis (15); however, CA-199 levels significantly decrease following surgery, indicating the effectiveness of HIFU combined with chemotherapy in controlling disease progression.

## Conclusion

HIFU is currently regarded by the medical community as a safe, effective, and innovative treatment modality with significant potential for tumor therapy. In addition to its direct impact on tumors, HIFU also has synergistic effects when combined with chemotherapy. The integration of HIFU with surgery, chemotherapy, and radiotherapy has yielded favorable clinical outcomes in the comprehensive management of tumors, thus presenting a valuable new therapeutic approach.

## Data Availability

The original contributions presented in the study are included in the article/supplementary material. Further inquiries can be directed to the corresponding author/s.
